# The vertebrate sialylation machinery: structure-function and molecular evolution of GT-29 sialyltransferases

**DOI:** 10.1007/s10719-023-10123-w

**Published:** 2023-05-29

**Authors:** Anne Harduin-Lepers

**Affiliations:** Univ. Lille, CNRS, UMR 8576 - UGSF - Unité de Glycobiologie Structurale et Fonctionnelle, F-59000 Lille, France

**Keywords:** Sialic acid, Sialyltransferase, Structure-function, Evolution, Enzyme activity

## Abstract

Every eukaryotic cell is covered with a thick layer of complex carbohydrates with essential roles in their social life. In Deuterostoma, sialic acids present at the outermost positions of glycans of glycoconjugates are known to be key players in cellular interactions including host-pathogen interactions. Their negative charge and hydrophilic properties enable their roles in various normal and pathological states and their expression is altered in many diseases including cancers. Sialylation of glycoproteins and glycolipids is orchestrated by the regulated expression of twenty sialyltransferases in human tissues with distinct enzymatic characteristics and preferences for substrates and linkages formed. However, still very little is known on the functional organization of sialyltransferases in the Golgi apparatus and how the sialylation machinery is finely regulated to provide the *ad hoc* sialome to the cell. This review summarizes current knowledge on sialyltransferases, their structure–function relationships, molecular evolution, and their implications in human biology.

## Introduction

My scientific career in the sialobiology field began in 1986 as a PhD student under the supervision of Professor André Verbert at Lille University, France. The aim of my studies was to understand how charged molecules like the sugar-nucleotides could reach the site of glycosylation within the Golgi apparatus. At that time, I studied transport of sialic acids from the cytosol to the Golgi apparatus, where the sialyltransferases were known to be located using permeabilized cells [1] and microsomes preparations. Using radiolabeled CMP-Neu5Ac in these *in situ* models, I could show the existence in the Golgi membranes of an antiporter allowing CMP/CMP-Neu5Ac exchange. In 1989, I had the great opportunity as an Erasmus student to spend six weeks in Professor Roland Schauer’s laboratory in Kiel. R. Schauer was professor of Biochemistry and director of the Institute of Biochemistry at the Christian-Albrechts-Universität Kiel. He was studying the biosynthetic pathways leading to other natural sialic acids like Neu5Gc or *O*-acetylated derivatives. He and his co-workers had elucidated some aspects of CMP-Neu5Gc biosynthesis showing that the major mechanism was hydroxylation of CMP-Neu5Ac thanks to a cytosolic hydroxylase. In his lab, I conducted competition studies to show that CMP-Neu5Ac and CMP-Neu5Gc shared the same carrier molecule involving CMP in mouse Golgi vesicles [2]. In addition, we showed that the factor determining the higher amount of Neu5Gc in mouse liver glycoproteins (95%) compared to rat liver glycoproteins (5%) was not the antiporter nor the sialyltransferases, but the mouse hydroxylase activity present in the cytosol [3]. This first meeting with Pr R. Schauer and his wife Elfriede was decisive for the rest of my scientific carrier as it definitely oriented it in the field the sialic acid biology and more specifically on sialyltransferases and how this terminal sialylation step is regulated in vertebrates. Pr R. Schauer has been for me a major guide in the sialobiology field following my work with always a lot of enthusiasm and kindness, and most of this work would not have been possible without the fruitful supporting and stimulating interactions I have had with him over the years.

In this review dedicated to the late Pr. R. Schauer, I summarized the various research developments in vertebrate sialic acid biology and focused on the recent insights gained on the structure-function studies of sialyltransferases.

## Structural diversity and distribution of Sialic acids in Metazoa

### Sialic acid structures

**Fig. 1:**
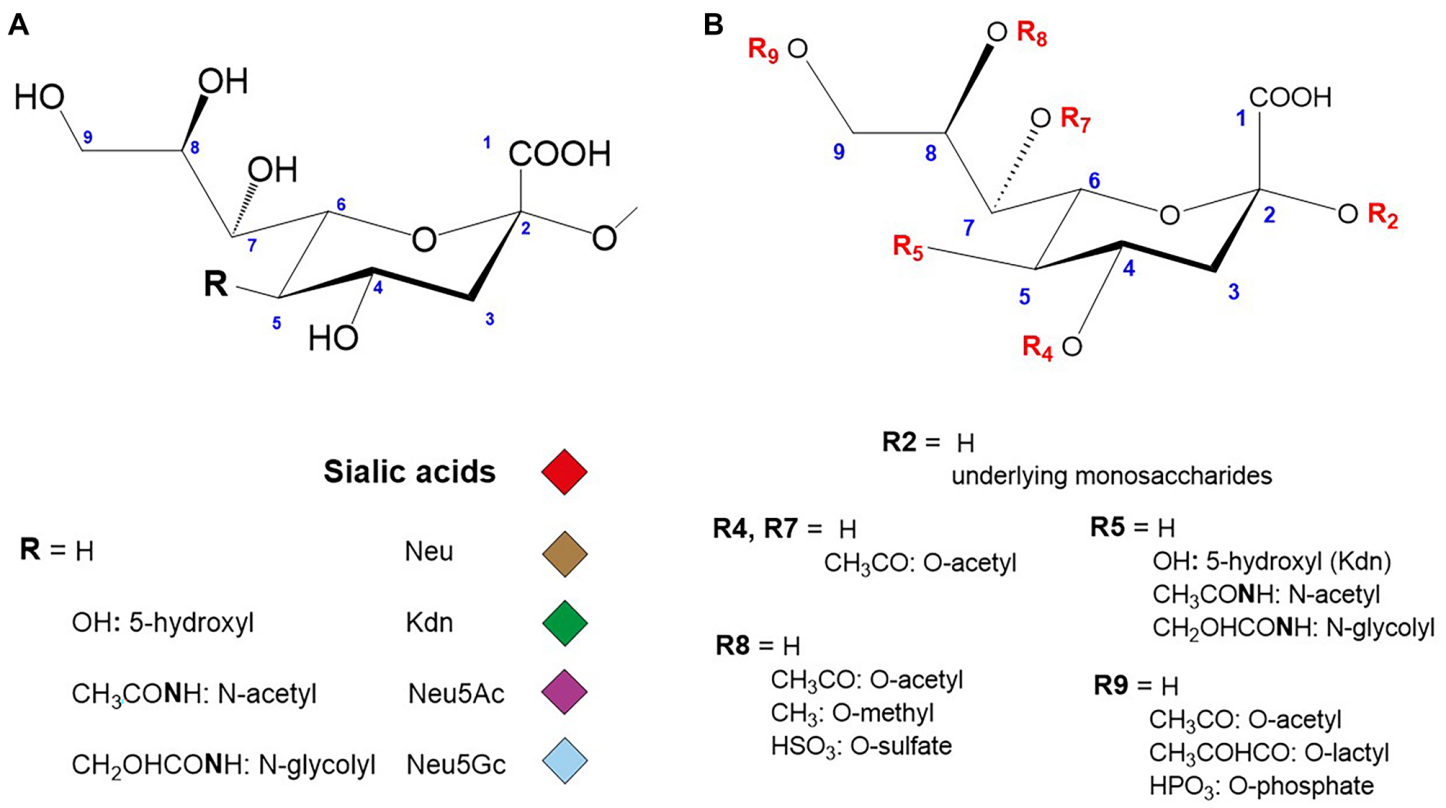
**A** Major sialic acid structures encountered in vertebrates. Sialic acids are illustrated with colored diamonds according to the SNFG nomenclature [176, 177]. **B** Modifications of sialic acids with *O*-acetyl, *O-*methyl, *O*-lactyl, *O*-phosphate and *O*-sulfate groups are depicted on each Sia carbon atom numbered in blue as described in [5]

Sialic acids (Sia) represent a highly diverse set of nine-carbon monosaccharides (Nonulosonic acids: NulOs), derivatives of neuraminic acid (Neu, 5-amino, 3,5-dideoxy-D-*glycero*-D-*galacto*-nonulosonicacid) all containing three functional groups: a carboxyl group at the C2 anomeric carbon, a glycerol-like three carbon side chain at the C6 carbon and an amino-acyl group or hydroxyl group attached to the C5 carbon (Fig. 1A) [4, 5]. Neu is almost not present in nature, but the derivatives of *N*-acetylneuraminic acid (Neu5Ac), *N*-glycolylneuraminic acid (Neu5Gc), and ketodeoxynonulosonic acid (Kdn, 3-deoxy-non-2-ulosonic acid) are the main Sia encountered. These Sia molecules can be substituted with glycolyl (Gc), acetyl (Ac), methyl (Me), lactyl, phosphate and sulfate groups generating a family over 50 structurally different members (Fig. 1B) [4, 6–8].

An additional level of structural diversity of these molecules results from the different glycosidic linkages formed between Sia (*e.g.* α-2,4, α-2,8, α-2,9 and α-2,5-*O*-glycolyl and the different degrees of polymerization (DP). DiSia (DP = 2), oligoSia (3 < DP < 7) or polySia (DP ≥ 8) that can reach 400 units [9, 10] have been described in vertebrates.

### Distribution of sialylated molecules in Metazoa

Sia have been described mainly in Deuterostoma, *i.e.* vertebrates, echinoderms, hemichordates and cephalochordates [4, 11, 12]. Although it has long been controversial, Sia are also reported in ecdysozoa protostomians like arthropods as *Drosophila melanogaster* (Dme) [13, 14] or *Galleria mellonella* [15], and in lophotrochozoa protostomians mollusks like the cephalopods *Octopus vulgaris* [16, 17], the gastropod *Arion lusitanicus* [18], or in pathogenic fungi such as *Candida albicans* [4, 19]. However, Sia are not found in plants, in archeabacteria nor in the ecdysozoa protostomia *Caenorhabditis elegans* [4] and thus show discontinuous distribution across evolutionary lineages [20]. Neu5Ac is also found in Prokaryota associated to glycolipids known as lipopolysaccharides (LPS) and of capsular polysaccharides of Gram-negative bacteria like the pathogen *Escherichia coli* K1, and a number of other NulOs are described in proteobacteria among which the 5, 7-diamino-3, 5, 7,9-tetradeoxy-D-*glycero*-D-*galacto*-nonulosonic acid (Legionaminic acid (Leg)) and the 5, 7-diamino-3, 5, 7, 9-tetradeoxy-L-*glycero*-L-*manno*-nonulosonic (Pseudaminic acid (Pse)) [4, 21].

The major Sia encountered in Deuterostoma is Neu5Ac. Neu5Gc shows a very unusual distribution across tissues and animal species; although it is also found in most deuterostomes like echinoderms (sea urchin and star fish), it was lost independently in various vertebrate branches and lineages. It is found in most placental mammals like horses, pigs and cows [22] with notable exceptions like humans, ferrets and monotremes like platypus [23]. It is not found in birds and reptiles [23–25] although low levels of Neu5Gc were described in the eggs and adult tissues of the green basilisk lizard [23]. Apart from the polyNeu5Gc found in the polysialylglycoprotein (PSGP) from salmonid eggs [26], only low levels of Neu5Gc were found in most tissues of fish and frogs although Neu5Gc was not found in *Rana esculata* [27]. Of particular note, Neu5Gc is usually not detected in vertebrate brain and the biological relevance of this observation is still not well understood [28–30]. Kdn harboring a hydroxyl group at C5 is less abundant in mammals and most abundant in cold-blooded vertebrates like amphibians and fish [31, 32]. It is observed in salmonid and amphibian eggs [33–35]. Free and conjugated forms of Kdn have been reported in human cancers [36, 37] as a result of high Man-6P and Fru-6P levels [38]. Kdn-glycoconjugates have been reported in microalgae such as *Emiliania huxleyi* [39] and *Prymnesium parvum* [40].

**Fig. 2:**
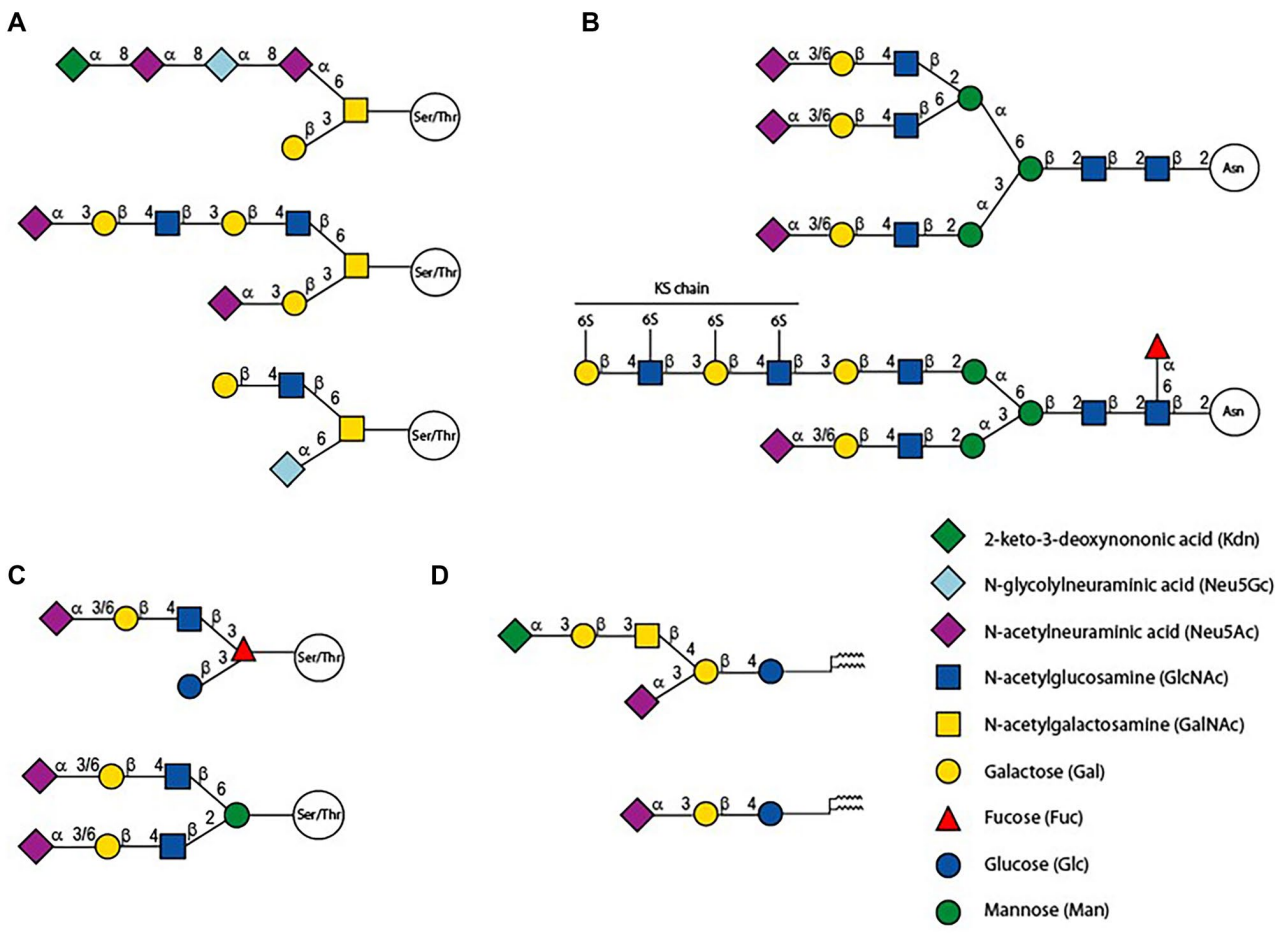
Examples of sialylated structures found on (**A**) GalNAc- or mucin-type *O*-glycans of glycoproteins like the fish polysialylated glycoprotein (PSGP), the core 2 mucin type *O*-glycans described in human colon and the bovine submaxillary mucin (BSM) (**B**) *N*-glycans of proteins like the bovine fetuin and the sialylated Keratan-sulfate (KS-I) found on aggrecan [178] (**C**) Fuc- and Man-type *O*-glycans of secreted matricellular proteins like Notch and α-dystroglycan [179] (**D**) glycolipids of the ganglioside series like the Kdn-containing GD1a from rainbow trout testis [180] and GM3. Sialylated glycans are represented according to the SNFG nomenclature [176, 177]

Sias are found at the outermost ends of glycoproteins and glycolipids constituting the sialome of an organism. As illustrated in Fig. 2, Sia are glycosidically linked to either the 3- or 6-hydroxyl groups of β-D-galactopyranosyl (Gal) residues, or to the 6-hydroxyl group of β-D-*N*-acetylglucosaminyl (GlcNAc) or of β-D-*N*-acetylgalactosaminyl (GalNAc) residues and can even form di-, oligo-, or poly-Sia chains *via* their 8-hydroxyl group and terminate with a Sia linked *via* the 8-or 9-hydroxyl group. Sialylated molecules can be secreted or attached to cell membranes leading to a huge diversity of sialoglycoconjugates at the cell surface known as sialome [41]. The sialome is highly diverse and variable according to the organism studied and even closely related animals such as a mouse and a rat, which diverge 25 million years ago (MYA), show a different sialome. The simplest sialome profiles are found in human and the evolutionary changes observed in primates Sia biology are thought to have major implications for human biology and diseases [38, 42–44].

### Sia implication in human physiology and pathologies

Due to the terminal position at the non-reducing end of glycans on cell-surface lipids and proteins, and the polar and charged nature of Sia, sialylated molecules play fundamental roles in many physiological processes and in the social life of vertebrate cells. They are pivotal molecules related to molecular and cellular recognition in most biological systems and processes like polySia-NCAM during embryonic development or polySia-PSGP during fertilization, and they serve as a molecular signal to modulate innate immunes responses [45]. Known intrinsic vertebrate sialic acid–binding proteins include factor H, selectins and sialic acid-binding immunoglobulin-like lectins known as Siglecs [5, 46–51]. Sia also serve as ligands for receptors mediated interactions in host–pathogen recognition involving bacteria and viruses like *Helicobacter pylori* [52] or influenza virus A [53] and coronavirus [54] reviewed recently [55]. The huge variety of biological functions of Sia reflect their structure diversity [56, 57]. In addition, their dynamic changes and aberrant expression are associated with several pathologies including neurological disorders like schizophrenia, and tumor metastasis [58, 59].

It has long been known that the cell surface of cancer cells is covered with Tumor Associated Carbohydrate Antigens (TACA) including sialylated molecules and hypersialylation of tumor cell surface glycoconjugates is a well-established hallmark of cancer with fundamental implication in tumor growth, metastasis, immune evasion and drug resistance [60, 61]. Among these sialylated TACA, the Thomsen Freidenreich antigens sialyl-Tn (CA72-4: sTn, Neu5Acα2,6GalNAc), sialyl-T (sT, Neu5Acα2,3Galβ1,3GalNAc) are truncated *O*-glycans frequently observed on proteins in breast cancer cell lines, and bladder cancer. Other sialylated structures that show increased levels in cancer include the serological cancer-biomarker sialyl-Lewis a (CA19.9; sLe^a^: Neu5Ac α2,3Galβ1,3[Fuc α1,4]GlcNAc) and the selectin ligand sialyl-Lewis x (sLe^x^: Neu5Ac α2,3Gal β1,4[Fuc α1,3]GlcNAc) which correlate with poor patient survival [62–65], the α2,3- or α2,6-linked sialic acid to *N*-acetyllactosamine (SLN) on *N*-glycosylproteins like cell receptors [66]. Solid tumor cells express hypersialylated neural cell adhesion molecule (NCAM), neuropilin-2 (NRP-2), or synaptic cell adhesion molecule 1 (SynCAM 1) to protect themselves from the immune effector cells [67]. Gangliosides like GM3, GM2, GD3 and GD2 are found in normal human tissues and are overexpressed in different cancer like lung cancer, melanoma, neuroblastoma, and breast cancer, in which they mediate cell proliferation, tumor growth and cancer cell migration [68]. In addition, cancer patients have been reported to express ‘Hanganutziu–Deicher’ antibodies that recognize gangliosides carrying the non-human Sia Neu5Gc, also detected in human tumors [69–72]. These sialylated TACA represent useful prognostic and diagnosis tools.

These sialylated TACA possess immunomodulatory properties and dendritic cells (DCs) are capable of sense them and stimulate naive T cells, thereby initiating the adaptive immune response [67, 73]. Siglecs are a family of 15 human cell surface receptors [74] that are primarily expressed by cells of the immune system, with the exception of most T cells. Siglecs recognize sialylated TACA expressed by tumor cells and mediate immunoregulatory signals both on myeloid and lymphoid immune cells [75, 76], suppressing T cell responses [77] and NK cytotoxicity [78], inducing the expression of transforming growth factor (TGF) by macrophages [79] modulating the immune response to cancer.

To explain these observations and gain a better understanding of these dynamic changes of the sialome, several questions remain to be answered like: how this diversity of the sialome is regulated? What is the sialylation machinery involved in the biosynthetic pathway of sialic acid molecules?

## Human sialyltransferases: from genes to sialylated products

Human sialyltransferases represent a large set of inverting glycosyltransferases grouped in the glycosyltransferase GT-29 family of the CAZy database (CAZy, available at http://www.cazy.org/), which classifies all enzymes active on carbohydrates [80]. They are Leloir-type metal-independent enzymes catalyzing the transfer of Sia from the nucleotide-activated sugar donor cytidine 5’- monophosphate-β-Neu5Ac (CMP-β-Neu5Ac) to the non-reducing end of a growing carbohydrate chain linked to a protein or a lipid generating α-linkages. These enzymes exhibit a high fidelity of reaction and a high acceptor specificity with no side activity like ST3Gal V generating GM3 ganglioside only (Table 1).

**Table 1:**
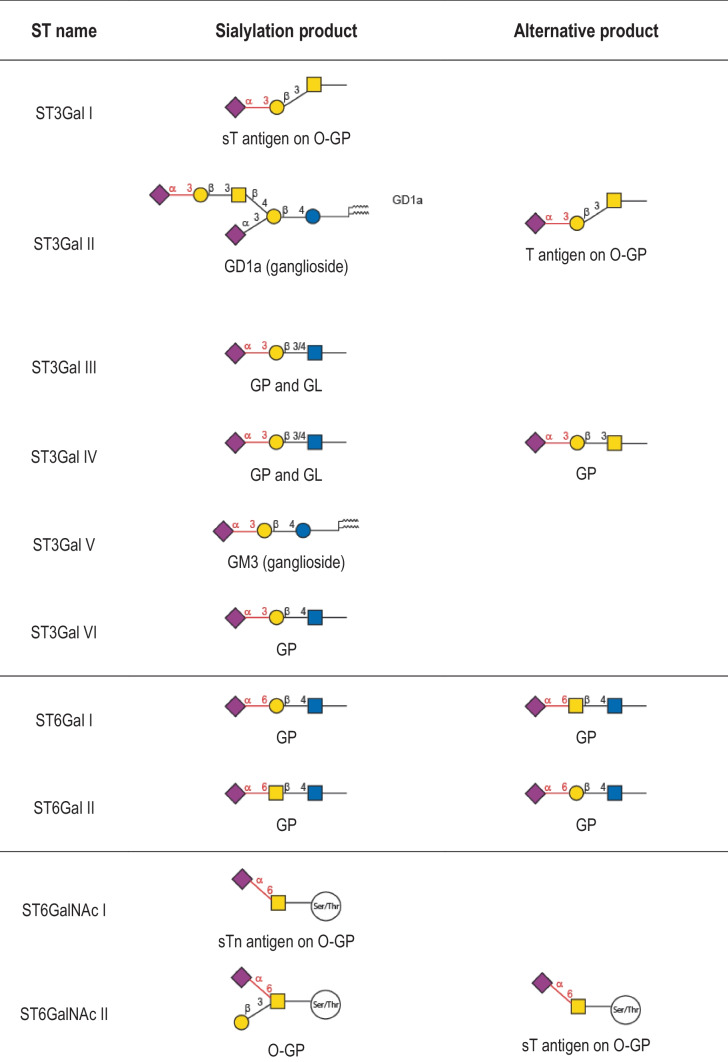

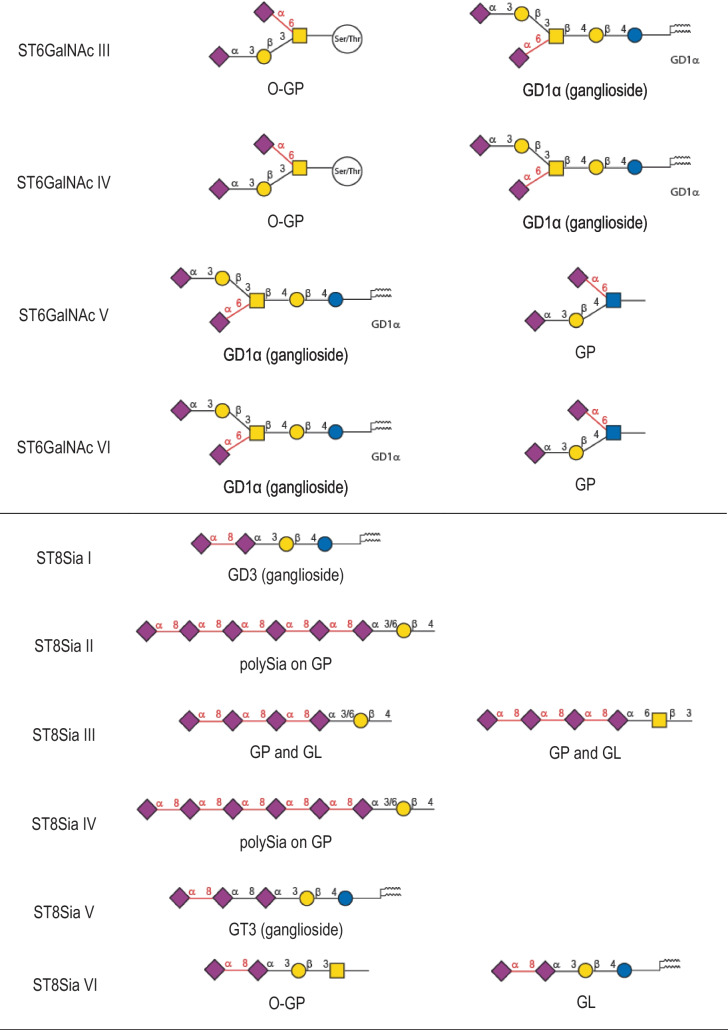
Human sialyltransferase enzymatic specificities. The twenty human sialyltransferases are listed and the preferred and secondary sialylated product formed is indicated

On the other hand, they have a relatively more relaxed donor substrate specificity [81, 82], although their donor substrate specificities have been less explored. CMP-Neu5Gc, CMP-Neu5,9Ac2 [83], and CMP-Kdn [84] have shown to be acceptable donor substrates by some mammalian sialyltransferases like fish polysialyltransferase [85].

### Human sialyltransferases genes

Twenty sialyltransferase genes have been identified in the human genome [82, 86–88]. Studies of the genomic organization and chromosomal assignment of the human genes have shown that the human sialyltransferase genes are polyexonic and widely dispersed in the genome on several chromosomes. Comparison of exon/intron boundaries and exons sizes of sialyltransferase genes have shown that they have a similar genomic structure delineating groups of genes likely originating from a common ancestor [82, 87]. Two *ST6GAL* genes named *ST6GAL1* and *ST6GAL2* according to the HUGO Gene Nomenclature Committee (HGNC), six *ST6GALNAC* genes (*ST6GALNAC1*-*6*), six *ST8SIA* genes (*ST8SIA1-6*) and six *ST3GAL* genes (*ST3GAL1-6*) were described in the human genome (https://www.genenames.org) [86, 88]. At least three pseudogenes were identified in ENSEMBL, *ST3GAL1P1* on chromosome 4, *ST6GALNAC2P1* on chromosome 2 and *ST6GALNAC4P1* on chromosome 13 that could be retro-transposed or remnants of an ancestral gene.

### Structure-function studies and biochemical characterization of human sialyltransferases

In the nineties, thanks to the molecular biology developments, most of the human sialyltransferases cDNA were cloned allowing the production of recombinant proteins and their functional characterization [82, 87, 89]. Biochemical characterization of a number of human sialyltransferases was achieved using soluble truncated protein restricted to their catalytic domains *in vitro* assays, radiolabeled CMP-[^14^C]Neu5Ac as a substrate donor and synthetic acceptors substrates either purified glycoproteins or glycolipids, shedding light on their substrate specificity. They were classified into four families depending on the type of linkage formed in α-2,3, α-2,6 or α-2,8 and on the nature of the monosaccharide acceptor namely ST6GAL, ST6GALNAC, ST3GAL and ST8SIA and each member was named accordingly [81, 90].

The six protein members of the ST3GAL family (ST3Gal I to ST3Gal VI) catalyze the formation of a α2,3-linkage between Neu5Ac and terminal galactose (Gal) residues found on glycoproteins (Galβ1,3GalNAc on *O*-glycosylproteins (O-GP) and Galβ1,3/4GlcNAc on *N*-glycosylproteins (N-GP)) and glycolipids as illustrated in Table 1. ST6Gal I and ST6Gal II in the ST6GAL family catalyze the transfer of Neu5Ac residues to the hydroxyl group in C6 of a terminal Gal residue of the type 2 disaccharide Galβ1-4GlcNAc, and potentially to the *N*-acetylgalactosamine (GalNAc) residue of LacdiNAc disaccharide (GalNAcβ1-4GlcNAc) [91]. The recombinant human ST6Gal I shows a broad substrate specificity towards these Gal(NAc)β1-4GlcNAc bearing substrates, whereas ST6Gal II exhibits *in vitro* a more restricted substrate specificity towards a few Galβ1-4GlcNAc and GalNAcβ1-4GlcNAc bearing glycoconjugates [91]. The ST6GALNAC family comprises six different members (ST6GalNAc I to ST6GalNAc VI) that catalyze similar reactions using a GalNAc residue found on mucin-type O-GP as an acceptor monosaccharide (ST6GalNAc I, ST6GalNAc II and ST6GalNAc IV) or on glycolipids (ST6GalNAc III, ST6GalNAc V and ST6GalNAc VI) to form gangliosides of the α-series. Interestingly, ST6GalNAc I and ST6GalNAc II show a narrow acceptor specificity requiring a GalNAc residue *O*-linked to a peptide acceptor substrate [92–94] whereas ST6GalNAc V and ST6GalNAc VI show a broader specificity also catalyzing the transfer of sialic acid on a GlcNAc residue leading to the formation of disialyl lactotetraosyl-ceramide (Lc4), a precursor of disialyl-Lewis a (disialyl-Le^a^) [95]. The six enzymes of the ST8SIA (ST8Sia I to ST8Sia VI) mediate the transfer of Neu5Ac to the hydroxyl group in C8 of another terminal Neu5Ac residue forming α2,8-linkages found on glycoproteins and glycolipids (Table 1). ST8Sia I, ST8Sia V and ST8Sia VI are mono-α2,8-sialyltransferases, ST8Sia III is an oligo-α2,8-sialyltransferase and ST8Sia II and ST8Sia IV are polysialyltransferases.

The human sialyltransferases adopt the same topology as other Golgi-glycosyltransferases in the *trans*-Golgi and *trans*-Golgi Network: they are type II transmembrane proteins showing a short cytoplasmic tail (~ 10–15 aa) a unique trans membrane domain, an intermediate stem region and a large catalytic domain (~ 250 aa) oriented within the Golgi lumen [86]. The functional organization of these enzymes within the Golgi membranes and the influence of the Golgi environment are still not understood; yet seminal studies of Kellokumpu’s group indicated the impact of Golgi pH, and ions and redox homeostasis [96, 97] as well as the formation of Golgi-glycosyltransferase complexes [98, 99] in the correct final sialylation status of glycoconjugates [100]. In addition, some sialyltransferases like ST6Gal I undergo proteolytic cleavage by proteases like the β-secretase BACE 1 or signal-peptide peptidase-like 3 (SPPL3) and are found in biological fluids [101, 102]. These enzymes undergo a series of post-translational modifications along their biosynthesis such as *O*- and *N*-glycosylation, disulfide-bond formation, which modulate their proper folding, dimer assembly and are essential to their enzymatic activity [103]. Therefore, these membrane-bound Golgi enzymes are very difficult to produce in high yield in a recombinant and active form and many challenges thus remain in understanding their function at the molecular level. The recent developments using eukaryotic HEK293 cells [104, 105] and progress in the biotechnology of glycosyltransferases afforded by structure-based rational design and directed evolution approaches [106, 107] led to the generation of suitable amounts of these stereoselective human sialyltransferases amenable to structural and biochemical studies for the refinement of their kinetic properties [104, 105, 108–110].

**Fig. 3:**
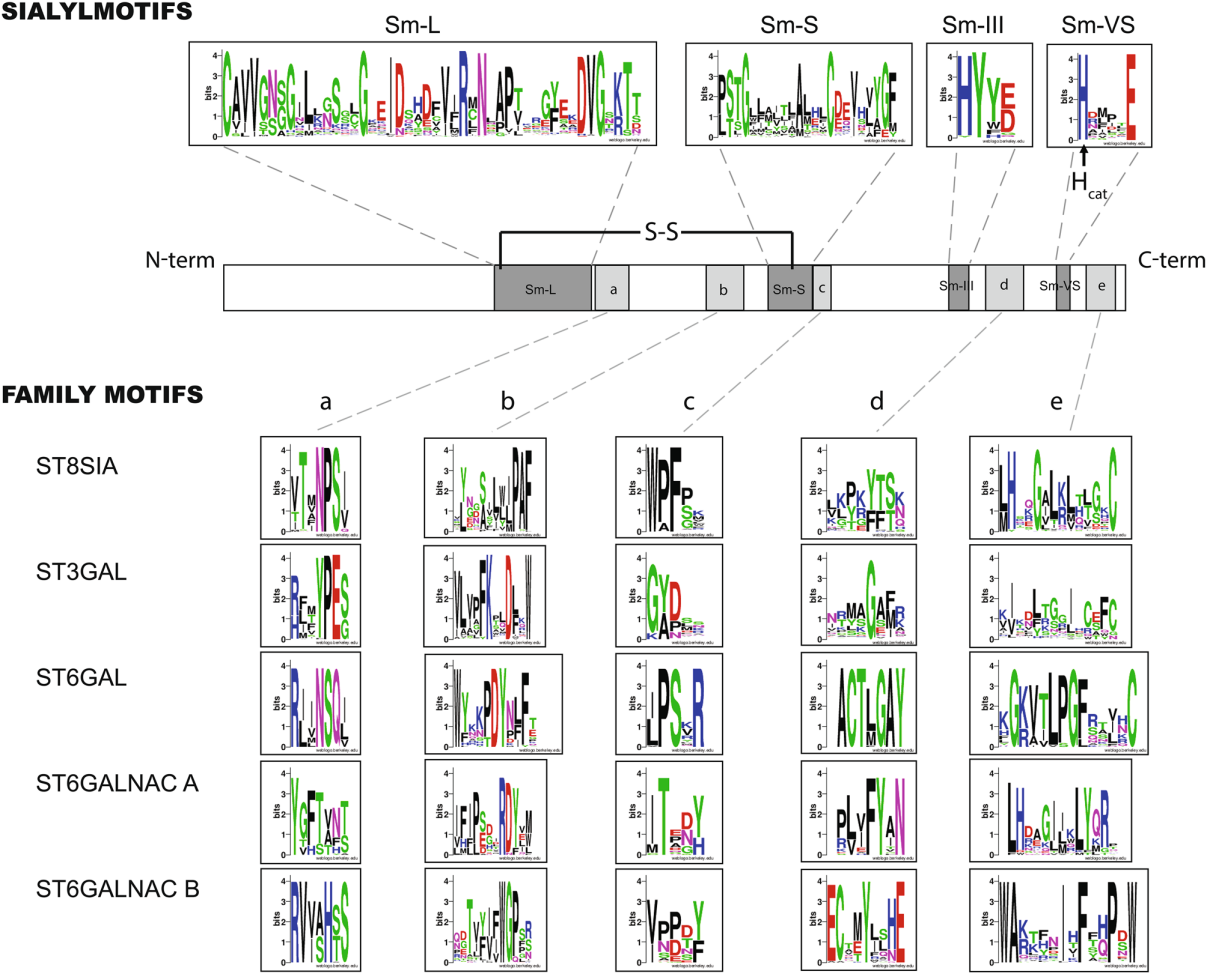
Schematic representation of the catalytic domain of the GT29 sialyltransferases. The upper part represents sequence logos of the sialylmotifs (Sm-L, Sm-S, Sm-III and Sm-VS) derived by the Berkeley Weblogo tool [181] after multiple sequence alignments of 308 representatives of each vertebrate subfamilies *i.e.* two ST6GAL, nine ST3GAL, six ST6GALNAC and nine ST8SIA subfamilies. The catalytic Histidine residue in the sialylmotif VS of GT29 sialyltransferases is indicated (H_cat_). The middle part indicates position of conserved motifs in the catalytic domain and the lower panel illustrates the family motifs (**a**–**e**). In the logos, one letter amino acid symbols are colored according to their chemical properties: polar amino acids (G, C, S, T, Y) are green, basic (K, R, H) are blue, acidic (D, E) are red, hydrophobic (A, V, L, I, P, W, F, M) are black and neutral polar amino acids (N, Q) are pink. The overall height of the stacks indicates the sequence conservation at a given position, while the height of symbols within the stack indicates the relative frequency of each amino acid at that position

**Fig. 4:**
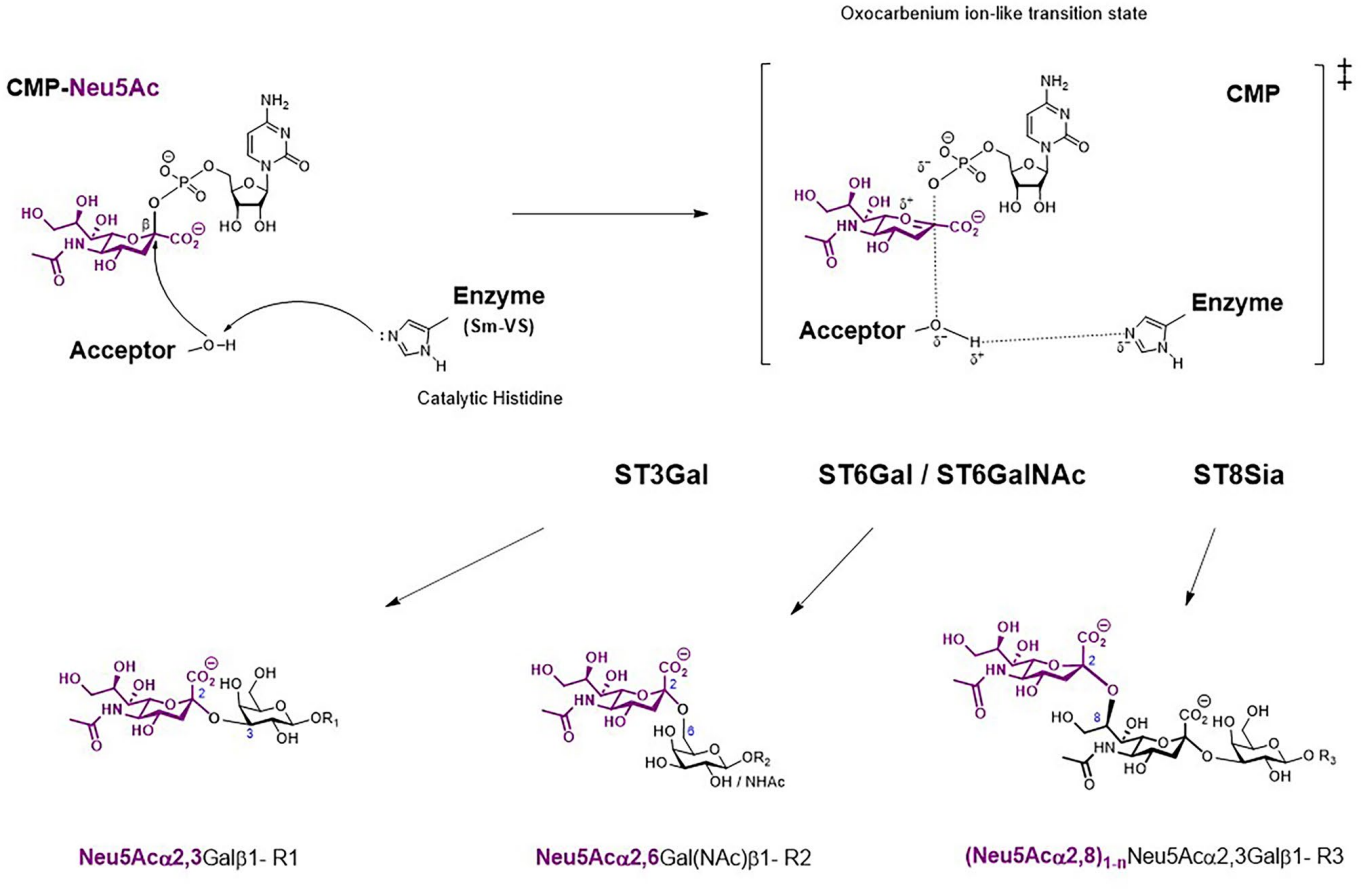
Schematic representation of the activities of inverting sialyltransferases. Human sialyltransferases use CMP-Neu5Ac and a SN2-like direct-displacement mechanism (upper part) to catalyze the transfer of Sia to a monosaccharide (Gal, GalNAc or Sia) acceptor (lower panel). The catalytic Histidine residue in the sialylmotif VS of sialyltransferases is indicated. An oxocarbenium ion transition state is formed and CMP acts as leaving group. Adapted from [123, 125, 147]

Despite very low sequence identity (< 30%) between the sialyltransferases subfamilies, comparative sequence-based analysis of the mammalian sialyltransferases have led to the discovery of conserved peptide motifs, the sialylmotifs Large (L), Small (S), III and Very Small (VS) [81] found in all the sialyltransferases of the CAZy GT-29 family (Fig. 3). Further site-directed mutagenesis strategies and structural approaches showed implication of the sialylmotifs in donor and acceptor binding and revealed a conserved histidine residue in the VS sialylmotif identified as the catalytic base [111–114] (Figs. 3 and 4). Highly conserved cysteines residues are found in sialylmotifs L and S forming intramolecular disulfide bounds [115, 116] that constrain sialyltransferase in a folded conformation. Further multiple sequence alignments of vertebrate sialyltransferase sequences led to the identification of family motifs named “a” to “e” characteristic of each vertebrate sialyltransferase family (Fig. 3), although their functional relevance still remain to be established [82, 117]. Also, specificity-determining positions (SDPs) *i.e*. the critical amino acids determining their functional specificity were determined for the ST3GAL and ST6GAL families [118, 119]. These amino acid positions often play critical roles as they are involved in the molecular mechanisms ensuring functional diversity. Finally, analysis of evolutionary co-mutations identified pairs of contacting and coevolving amino acid residues in the ST3GAL family likely important to maintain protein function [118].

Only three human sialyltransferases ST6Gal I [120, 121], ST6GalNAc II [105], ST8Sia III [122] and two additional vertebrate sialyltransferases rat ST6Gal I [123] and porcine ST3Gal I [114] were crystallized up to now with or without (apoenzyme form) their donor/acceptor substrates (Table 2). The GT-29 sialyltransferases are thought to use a single-displacement SN2-like mechanism (Fig. 4) and to adopt a modified GT-A fold structure called GT-A variant 2 comprised of the sialylmotifs L, S, III and VS, which lie in the core of the Rossmann-fold scaffold to assemble a metal-independent CMP-Neu5Ac binding site using adjoining proximal loop regions [82, 86, 111, 124, 125]. Interestingly, sialyltransferase structures show distinct binding-site architectures and significant differences in the amino acid residues predicted to interact with the glycan acceptor substrate [124].

**Table 2:** Crystal structures of sialyltransferases in the Protein Data Bank (PDB)

**Sialyltransferase**	**family**	**aa range**	**ligand**	**3D-structure in PDB**	**Organism**	**reference**
ST6Gal I	ST6GAL	89–406	BMA, CTN, GAL, MAN, NAG	4JS1	human	[123]
			3MA, C, FUL, GAL, MAN, NAG	4JS2		
ST6Gal I	ST6GAL	132–406	_	6QVS	human	[120]
			NCC	6QVT		
ST6GalNAc II	ST6GALNAC	66–374	NAG	6APJ	human	[105]
			C5P, NAG	6APL		
ST8Sia III	ST8SIA	81–380	BMA, CDP, FUC, NAG	5BO6	human	[122]
			FUC, CTP, NAG	5BO7		
			MAN, FUC, BMA, NAG	5BO8		
			GS, FUC, GAL, CSF, NAG, SIA	5BO9		
ST8Sia III	ST8SIA	81–380	NAG, BMA, FUC	5CXY	human	Not published
ST3Gal I	ST3GAL	46–343	_	2WML	porcine	[114]
			A2G, C5P, GAL	2WNB		
			A2G, GAL	2WNF		
ST6Gal I	ST6GAL	95–403	NAG	4MPS	Rat	[123]

Abbreviations of ligands are: unliganded (–), donor substrates are CMP-Neu5Ac (NCC), CMP (C or C5P), CDP, CDP-3-F-Neu5Ac (CSF), 4-amino-1-β-D-ribofyranosyl-2(1H)-pyrimidinone (CTN) and CTP. The acceptor substrates are *N*-acetyl-2-deoxy-2-amino-galactose (A2G), β-D-mannose (BMA), α-fucose (FUC), β-L-fucose (FUL), β-D-galactose (GAL), α-D-mannose (MAN), N-acetyl-D-glucosamine (NAG), 2-(acetylamino)-2-deoxy-6-O-sulfo-β-D-glucopyranose (NGS) and sialic acid (SIA)

### Variable expression of sialyltransferases in cancer

Hypersialylation of cancer cells and increased sialylated TACA described above result from dysregulation mainly at the transcriptional level involving alternative splicing and promoter utilization leading to differential sialyltransferases expression in various types of malignancies as broadly reviewed this past decade [126–130]. The main cancer associated sialyltransferases include ST3Gal I and ST3Gal II driving the expression of sT antigen and GD1a and GT1b gangliosides, which are overexpressed in different types of malignancies [131–133], ST3Gal III, ST3Gal IV and ST3Gal VI involved in the synthesis of sLe^a^ and sLe^x^ antigens and overexpressed in gastric carcinoma [134, 135], ST6Gal I contributing to the formation of Sia6LacNAc (SLN) in colon, stomach and ovarian cancers [136, 137], ST6GalNAc I and ST6GalNAc II generating sTn and sialyl-6-T antigens in breast and colon cancer and gastrointestinal tissues [93, 138–140], ST8Sia II forming polySia chains in small cell lung cancer and neuroblastoma [141, 142]. As mentioned before, changes in the Golgi environment associated with tumorigenesis like hypoxia, redox homeostasis or pH also impact sialyltransferases expression, assembly and localization in the Golgi apparatus [96, 97, 100, 143]. These sialyltransferases represent potential biomarkers and treatment targets.

The development of cell-permeable, non-toxic sialyltransferase specific small-molecule inhibitors is much needed and represents a field of intense investigations largely reviewed these past years [61, 76, 127, 129, 144–151]. Natural products such as soyasaponin I, ginsenosides and lithocholic acid and derivatives were shown to reduce sialylation modifying invasive behavior of tumor cells [152–155]. So far, the only one known pan-sialyltransferase inhibitor is the peracetylated sialic acid glycomimetic P3F_ax_-Neu5Ac [156] and encapsulated into tumor-targeting nanoparticles, 3Fax-Neu5Ac-related drugs were shown to impair adhesion, migration and delays tumor growth *in vivo* [157, 158]. The most potent sialyltransferase inhibitors to date are those mimicking the transition state of the sialylation process based on activated form of CMP-Neu5Ac (Fig. 4) [159, 160] including carbamate or triazole linker as an isosteric replacement for the phosphodiester to improve pharmacokinetic properties [161, 162]. In addition, the development of new methodologies like high-throughput screening (HTS) and rapid and sensitive biochemical assays [108, 163] exploiting sialyltransferase promiscuity towards artificial Sia or electrochemical biosensing platform [164] also contributed to open new avenues of drug discovery. Although some advances in sialyltransferase inhibitors have been achieved these past years, the field still suffers from many limitations: i) only a handful 3D-structure of sialyltransferases exist hindering the structure-based design of inhibitors targeting other sialyltransferases; ii) assessment of the selectivity of inhibitors is limited to the most studied and frequently targeted sialyltransferases ST3Gal I and ST6Gal I; iii) cell-based assays are limited and almost nothing is known on the cytotoxicity, cell uptake and target specificity of the newly developed small-molecule inhibitors. Therefore, advances in new approaches and methodologies for specific sialyltransferase targeting and cancer-specific delivery are still much required.

## Origin and evolution of GT-29 sialyltransferases and sialic acid pathway in Eukaryota

**Fig. 5:**
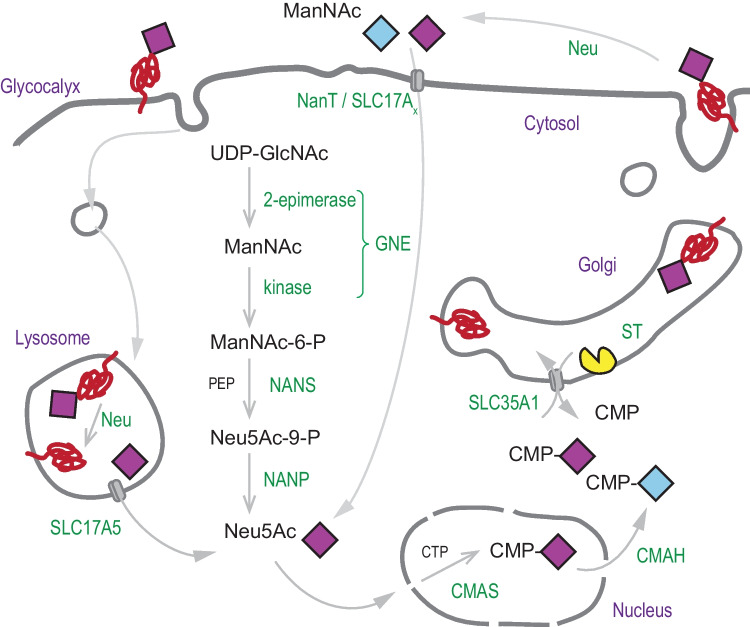
Illustration of the sialic acid pathway in cells. Sialic acid (Neu5Ac) is synthetized in the cytosol from UDP-GlcNAc in several steps involving GNE (UDP-GlcNAc 2-epimerase/ManNAc kinase, the two enzymatic domains are fused in Deuterostoma), NANS (Neu5Ac-9-phosphate synthetase or *N*-acetylneuraminate lyase) with PEP (phosphoenolpyruvate) and NANP (Neu5Ac-9-phosphate phosphatase). Exogenous sources of Sia come from the activity of Neu (sialidases 1–4), the lysosomal transporter sialin (SLC17A5) or from NanT/SLC35Ax the hypothetical plasma membrane transporter of Sia. Sias are used in the nucleus by CMAS (CMP-Sialic acid synthetase) with CTP (cytidine triphosphate) to form CMP-Neu5Ac, which can be used in the cytosol by CMAH (CMP-Neu5Ac hydroxylase) to form CMP-Neu5Gc and by SLC35A1 the Golgi CMP-sialic acid antiporter. Within the Golgi lumen, the various ST (GT29 sialyltransferases) uses CMP-Sia to sialylate glycoproteins and/or glycolipids)

**Fig. 6:**
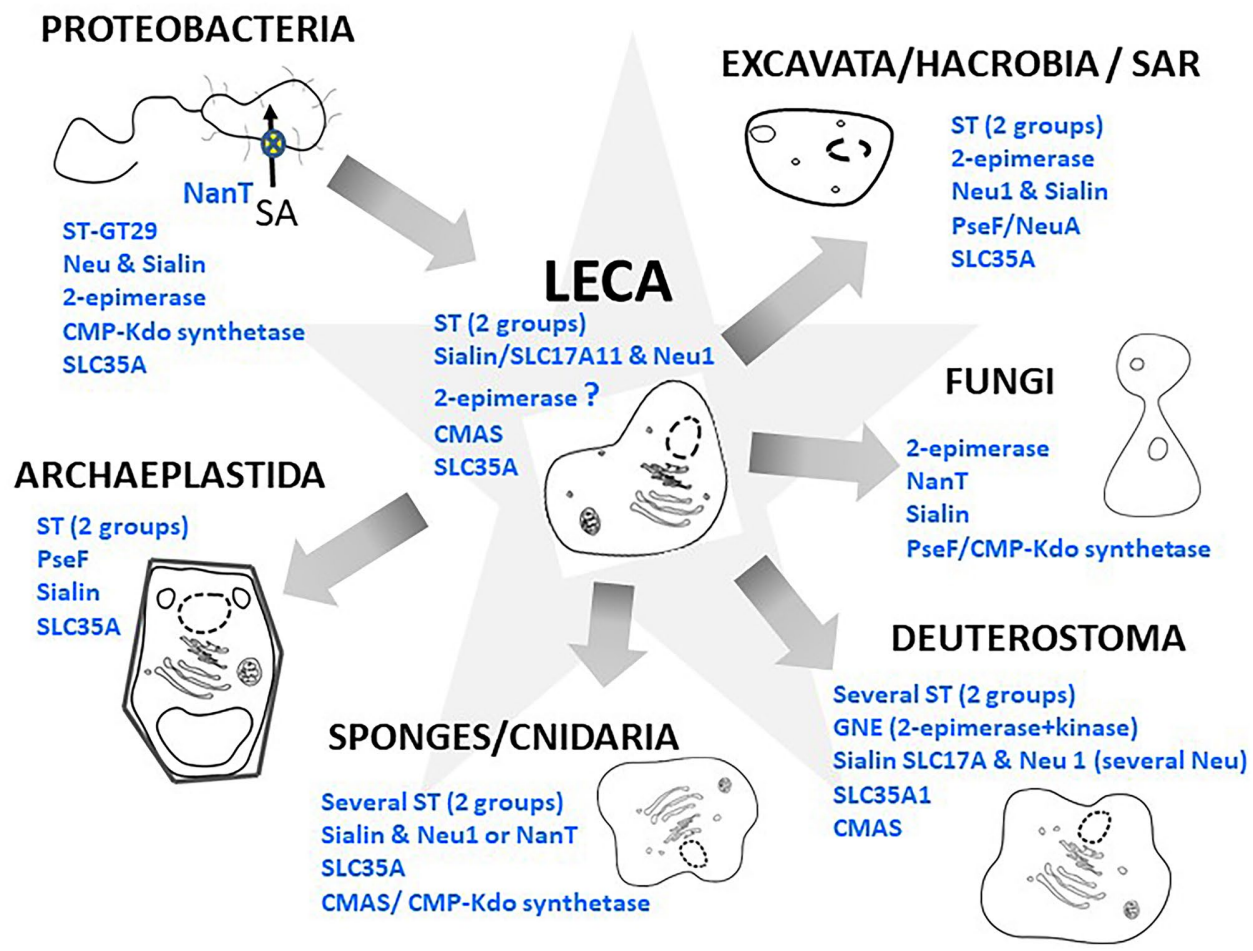
Origin and evolution of the sialylation machinery in eukaryotes. This schematic illustrates the findings of the Petit et al. 2018 study [165] reporting on the distribution of the major actors of the sialic acid pathway in various eukaryotic phyla (Excavata, Hacrobia and SAR, and in the Opisthokontha Fungi, Deuterostoma, sponges and Cnidaria) and in the Last Eukaryotic Common Ancestor (LECA). LECA possessed an exogenous source of Sia through the existence of sialidases (the lysosomal Neu1 and an as yet uncharacterized sialidase) in addition to transporters of the SLC17A family (SLC17A5 known as sialin and SLC17A11 a less specific transporter of glutamate, glucuronic and sialic acids). LECA also harbored an endogenous source of sialic acid using ManNAc as an initial substrate and Man-kinase, NANS and NANP enzymes. In the cytosolic compartment of LECA, the CMAS enzyme could activate the sialic acid molecule into CMP-sialic acid, which was then translocated into the Golgi compartment through the use of the transporter SLC35A1 and transferred to glycoconjugates by GT-29 sialyltransferases likely originating from α-proteobacteria

Evolutionary studies of the GT-29 sialyltransferases indicated a patchy distribution of these enzymes in eukaryotes [165]. In this recent study, GT-29 sialyltransferase-related sequences were search in the three domains of life including the five main eukaryotic branches. Nineteen were identified in protists, 30 in Archaeplastida, 106 in Opisthokonta, one in Amoebozoa, and one in Archaea and 23 in Alpha-, Gamma- and Epsilon-Proteobacteria. Although widespread among the three domains of life, no GT-29-related sequence could be identified in the eukaryotic branches of Excavata nor in Rhizaria, whereas an expansion of these sequences was noted in the prasinophyte *Bathycoccus prasinos* and in the sponge *Oscarella carmela* suggesting that the sialylation pathway was more ancient than anticipated. Comparative sequence-based analysis of these GT-29-related sequences in their informative region (92 aa in the catalytic domain) indicated the presence of conserved amino acid residues in the four sialylmotifs important to maintain structure and function of these enzymes. Molecular phylogenetic analyses and sequence similarity networks using Cytoscape led to the conclusion that the Last Eukaryotes Common Ancestor (LECA) already possessed two types of GT-29 sequences ST6Gal/ST6GalNAc III-VI and ST8Sia/ST6GalNAc I-II/ST3Gal likely inherited from a single sialyltransferase of proteobacteria [165], an horizontal gene transfer event that could be concomitant to the massive introduction of α-Proteobacterial genes in the first Eukaryotes that resulted in mitochondrial incorporation [165]. Along evolution, protists sialyltransferases likely conserved similar function to those of bacteria, whereas multicellular organisms have evolved new functions in cell–cell interaction through functional divergence in four distinct ancestral families described in invertebrates [88]. In addition, phylogenetic studies of key actors of the sialylation pathway were carried out [165]. Among the 60 glyco-genes known to be involved in sialic acid biology, some are endogenous to the cell and linked to Sia biosynthesis like the UDP-GlcNAc 2-epimerase/ManNAc kinase (GNE), the Neu5Ac-9-phosphate synthetase NANS (known as Neub in Bacteria and Archaea), the Neu5Ac-9-phosphate phosphatase NANP, some are involved in Sia use like sialyltransferases, the CMP-sialic acid synthase known as CSS or CMAS, the CMP-Neu5Ac hydroxylase (CMAH) and the CMP-sialic acid transporter SLC35A1 and the other are implicated in the catabolic pathway like sialidases (Neu) and transport of sialic acid like SLC17A5 (sialin) and the bacterial sialic acid transporter NanT (Fig. 5). These studies led to the conclusion that LECA possessed the ability to use either exogenous sialic acid molecules (presence of sialidases and transporters of the SLC17A family) or an endogenous sialic acid biosynthetic pathway involving a Man-kinase, NANS, NANP and CMAS in the cytosol to produce CMP-sialic acid and supply Golgi GT-29 sialyltransferases *via* the SLC35A1 transporter with their activated sugar-donor [165]. During eukaryotes evolution, this sialylation pathway was partially maintained or totally lost as illustrated in Fig. 6.

**Fig. 7:**
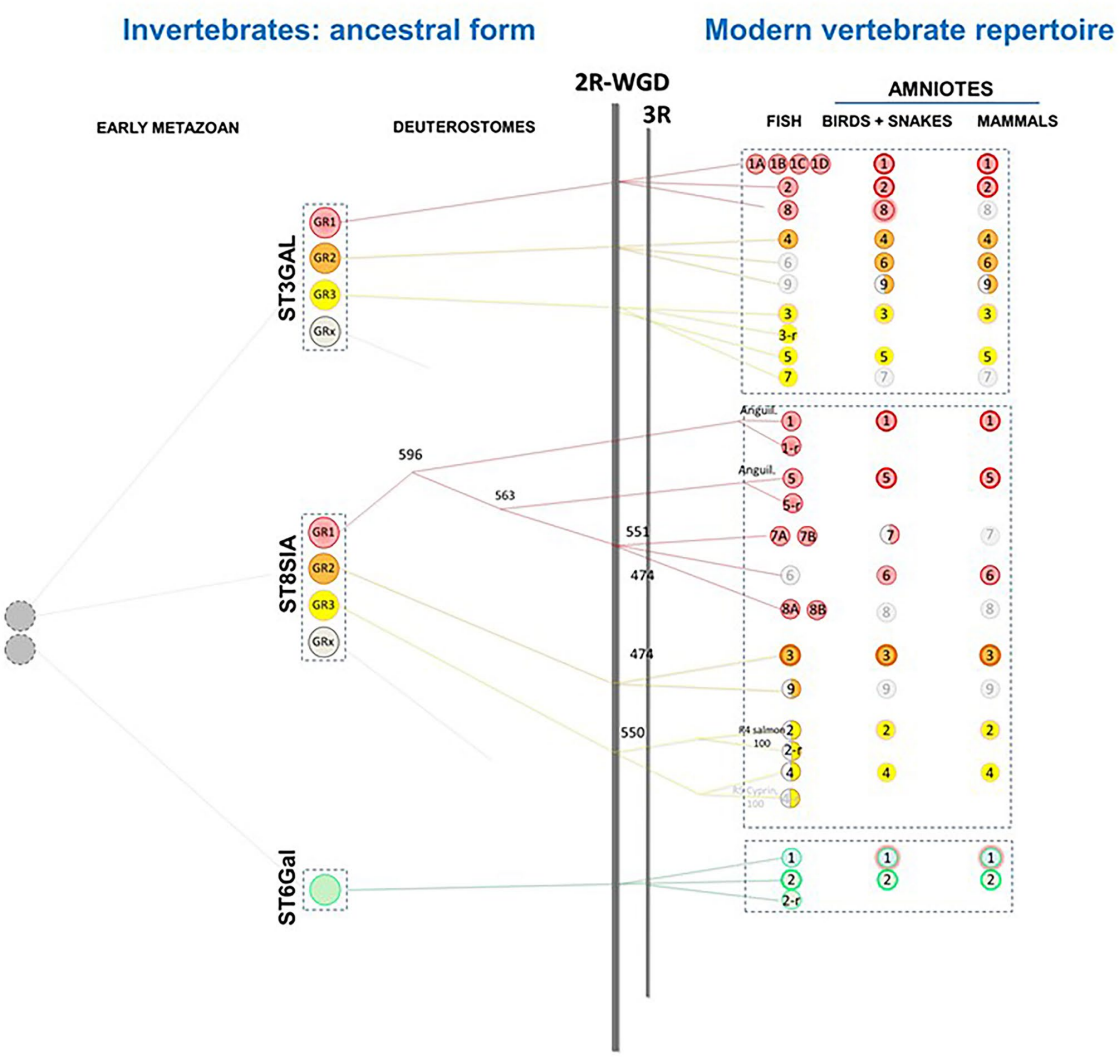
Illustration of the evolutionary scenario of GT-29 sialyltransferases in Metazoa. The two original groups of GT-29 sialyltransferases present in LECA gave rise to the four families ST3GAL, ST6GAL, ST6GALNAC and ST8SIA subdivided into groups of enzymes (GR) after gene duplications in Metazoa, which were maintained or not across evolution of Deuterostoma. Up to now, only ST8SIA, ST6GAL and ST3GAL evolutionary history could be reconstructed in Deuterostoma, therefore ST6GALNAC are not represented here [118, 119, 169, 170, 173]. A burst of innovations occurred at the base of vertebrates after two rounds of whole genome duplication events (WGD-R1 and WGD-2), after the Teleost-specific whole genome duplication event TGD and the species-specific genome duplication events (salmonid-specific 4R (Ss4R) and carp-specific 4R (Cs4R)) which were maintained or not in the various vertebrate branches

Seminal evolutionary studies of the GT-29 sialyltransferases identified in Metazoa showed the presence of ancestral gene families in the invertebrates and a burst of novelties in vertebrates through gene and genome duplication events and a first model of divergent evolution was proposed [82, 88, 119] (Fig. 7). This model highlighted the existence of ancestral sialyltransferase families in invertebrates and their evolutionary relationships with the vertebrate sialyltransferase sequences were established through sequence-based analysis showing that they are orthologous to the genes found in the last common ancestor of vertebrate. A single copy gene *st3gal1/2* was described in the tunicates *Ciona intestinalis* [88, 166] and *Ciona savignyi* [88, 167] and this gene was found to be ancestral to the vertebrate ST3Gal I and ST3Gal II subfamilies [88, 118]. Similarly, a single copy gene *st6gal1/2* (DSIAT) was described in the insects *Drosophila melanogaster* [13, 14, 88] that regulates the nervous system function in Drosophila [168]. This gene is ancestral to the two vertebrate subfamilies ST6Gal I and ST6Gal II [88, 119, 169]. Four groups of multiple copy *st8sia*-related genes were identified in the cephalochordates *Branchiostoma floridae*, ancestral to the vertebrate mono-α-2,8-sialyltransferases, oligo-α-2,8-sialyltransferases and poly-α-2,8-sialyltransferases subfamilies and the last group of ancestral genes named *st8siaex* disappeared in vertebrates [88, 170]. Finally, a single copy gene *st6galnac3/4/5/6* was identified in the sea urchin *Strongylocentrotus purpuratus* [88].

This model also highlighted the existence of several new vertebrate subfamilies. Comparative and functional genomics approaches were used to unravel the evolutionary relationships and fate of these newly described genes. Most of these new vertebrate subfamilies have arisen from the two whole genome duplication (WGD) events that took place at the base of vertebrate emergence, around 500 million years ago (MYA) for the WGD-R2 and ~ 555 MYA for WGD-R1, and after the teleost-specific WGD (TGD or Ts3R) that took place at the base of Actinopterygii ~ 320 MYA [118, 119, 169, 171–173]. These duplicated genes, paralogous to the one described in mammals were maintained or lost immediately after the WGD events or later on during vertebrate evolution, like *st3gal6, st3gal9* and *st8sia6,* which were lost in teleosts or *st3gal7* and *st8sia8* lost in tetrapods and *st3gal8* lost in mammals (Fig. 7). In addition, almost all the duplicated genes generated after the TGD were lost in the fish genomes with the exception of *st6gal2-r* and *st3gal3-r* genes conserved in the zebrafish genome [118, 119, 169]. Interestingly, several new ST8Sia subfamilies were identified resulting from fish specific WGD events beyond the Ts3R event like *st8sia2-r1* and *st8sia2-r2* resulting from the salmonid-specific 4R (Ss4R) ~ 100 MYA [174] or *st8sia4-r1* and *st8sia4-r2* resulting from the carp-specific 4R (Cs4R) [173, 175]. The recent enzymatic characterization of the salmonid polysialyltransferases ST8Sia IV, ST8Sia II-r1 and ST8Sia II-r2 pointed to a broader specificity towards CMP-sialic acid donors of the fish enzymes compared to their human orthologues (Decloquement et al. 2023, unpublished data).

Altogether, these studies of the functional diversification of vertebrate sialyltransferases provided a conceptual framework to understand sialylation evolution and explain why closely related species differ in their sialome. The evolutionary relationship of these sialyltransferase sequences could be established shedding light not only on their evolutionary history which was shaped mostly by the WGD and gene losses events, but also on the molecular function of newly described enzymes. Understanding how these proteins evolved will help addressing challenges of the future like those relating to the design and engineering of sialyltransferase with new molecular functions.

## Data Availability

Data and materials sharing not applicable – no new data generated.
